# Dimethyl 3,5-diethyl-1*H*-pyrrole-2,4-dicarboxyl­ate

**DOI:** 10.1107/S1600536811028352

**Published:** 2011-07-23

**Authors:** Gui-Fen Lu, Wen-Sheng Lin, Wei-Hua Zhu, Zhong-Ping Ou

**Affiliations:** a School of Chemistry and Chemical Engineering, Jiangsu University, Zhenjiang 212013, People’s Republic of China

## Abstract

The title pyrrole derivative, C_12_H_17_NO_4_, consists of a pyrrole ring with two diagonally attached meth­oxy­carbonyl groups and two diagonally attached ethyl groups. The two carbonyl groups are approximately in the same plane as the pyrrole ring, making dihedral angles of 3.50 (19) and 6.70 (19)°. In the crystal, adjacent mol­ecules are assembled into dimers in a head-to-head mode by pairs of inter­molecular N—H⋯O hydrogen bonds.

## Related literature

For applications of polysubstituted pyrroles, see: Brockmann & Tour (1995 ▶); Guilard *et al.* (2001 ▶); Trofimov *et al.* (2004 ▶). For related structures, see: Takaya *et al.* (2001 ▶). For background to complexes of pyrrole derivatives, see: Fan *et al.* (2008 ▶); Ou *et al.* (2009 ▶); Paixão *et al.* (2003 ▶); Yamamoto *et al.* (1986 ▶).
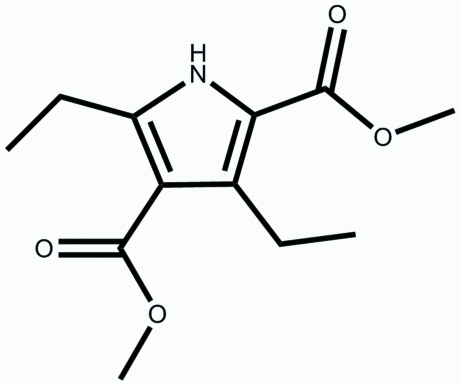


## Experimental

### 

#### Crystal data

C_12_H_17_NO_4_
*M* *_r_* = 239.27
Monoclinic, 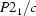

*a* = 4.4697 (7) Å
*b* = 14.616 (2) Å
*c* = 19.784 (3) Å
β = 90.467 (2)°
*V* = 1292.4 (4) Å^3^
*Z* = 4
Mo *K*α radiation
μ = 0.09 mm^−1^
*T* = 298 K
0.20 × 0.15 × 0.10 mm

#### Data collection

Bruker APEXII CCD area-detector diffractometer
Absorption correction: multi-scan (*SADABS*; Sheldrick, 2003 ▶) *T*_min_ = 0.982, *T*_max_ = 0.991
6296 measured reflections
2285 independent reflections
1977 reflections with *I* > 2σ(*I*)
*R*_int_ = 0.037

#### Refinement

*R*[*F* ^2^ > 2σ(*F* ^2^)] = 0.041
*wR*(*F* ^2^) = 0.118
*S* = 1.08
2285 reflections
155 parameters
1 restraint
H-atom parameters constrained
Δρ_max_ = 0.15 e Å^−3^
Δρ_min_ = −0.16 e Å^−3^

### 

Data collection: *APEX2* (Bruker, 2004 ▶); cell refinement: *SAINT-Plus* (Bruker, 2001 ▶); data reduction: *SAINT-Plus*; program(s) used to solve structure: *SHELXS97* (Sheldrick, 2008 ▶); program(s) used to refine structure: *SHELXL97* (Sheldrick, 2008 ▶); molecular graphics: *XP* in *SHELXTL* (Sheldrick, 2008 ▶); software used to prepare material for publication: *SHELXL97*.

## Supplementary Material

Crystal structure: contains datablock(s) global, I. DOI: 10.1107/S1600536811028352/pk2335sup1.cif

Structure factors: contains datablock(s) I. DOI: 10.1107/S1600536811028352/pk2335Isup2.hkl

Supplementary material file. DOI: 10.1107/S1600536811028352/pk2335Isup3.cml

Additional supplementary materials:  crystallographic information; 3D view; checkCIF report

## Figures and Tables

**Table 1:** Hydrogen-bond geometry (Å, °)

*D*—H⋯*A*	*D*—H	H⋯*A*	*D*⋯*A*	*D*—H⋯*A*
N1—H1*N*⋯O4^i^	0.85	2.07	2.8773 (15)	160

Symmetry code: (i) 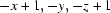.

**Fig. 1.**
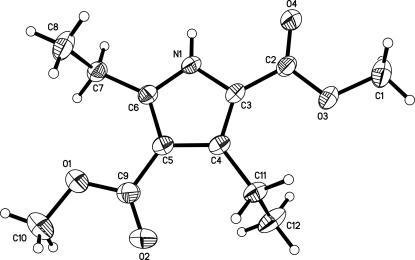
Molecular structure of the title compound. Displacement ellipsoids are drawn at the 30% probability level.

**Fig. 2.**
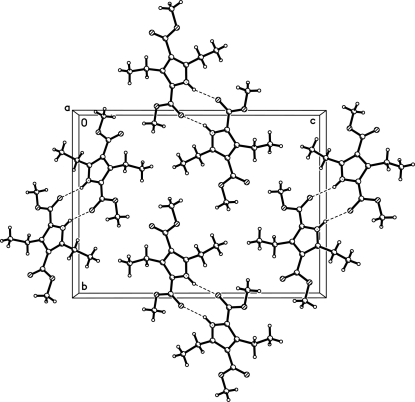
The two-dimensional supramolecular configuration, viewed down the a axis.

## supplementary crystallographic information

### Comment

Polysubstituted pyrroles have been paid much attention because of their wide application in the preparation of porphyrin (Trofimov *et al.*, 2004), corrole (Guilard *et al.*, 2001), and as monomers for polymer chemistry (Brockmann & Tour, 1995). In particular, 2-(alkoxycarbonyl)pyrrole derivatives have attracted intense interest in the design and synthesis of functional materials (Fan *et al.*, 2008).  The title compound was synthesized as a precursor to corrole compounds.

As shown in Fig. 1, the compound has a five-membered pyrrole ring as skeleton and four substituents.  Two diagonally related methoxycarbonyl groups and two diagonally related ethyl substituents are attached to the pyrrole ring. Pairs of intermolecular N1—H1n···O4^i^ (symmetry code i: -*x* + 1, -*y*, -*z* + 1) hydrogen bonds assemble adjacent molecules in a head-to-head manner, as shown in Fig. 2.  All bond distances are in the normal range for this type of compound, as reported by Yamamoto *et al.* (1986).

### Experimental

The title compound was synthesized from methyl 3-oxopentanoate by a Knorr-type reaction according to the method reported by Ou *et al.* (2009). Single crystals were grown from ethyl alcohol by slow evaporation.

### Refinement

All the non-hydrogen atoms were refined anisotropically by full-matrix least-squares calculations on F^2^.  All the H atoms expect H1n were placed in calculated positions with C—H distances of 0.93 and 0.96 /%A, and were refined using a riding model with *U*_iso_(H) = 1.2*U*_eq_(C). H1n was found in a difference map, and included using a riding model with a bond length restrained to 0.84 (1) Å and *U*_iso_(H) = 1.2*U*_eq_(N).

### Crystal data

**Table:**

C_12_H_17_NO_4_	*F*(000) = 512
*M**_r_* = 239.27	*D*_x_ = 1.230 Mg m^−^^3^
Monoclinic, *P*2_1_/*c*	Mo *K*α radiation, λ = 0.71073 Å
Hall symbol: -P 2ybc	Cell parameters from 3745 reflections
*a* = 4.4697 (7) Å	θ = 2.8–27.4°
*b* = 14.616 (2) Å	µ = 0.09 mm^−^^1^
*c* = 19.784 (3) Å	*T* = 298 K
β = 90.467 (2)°	Block, colorless
*V* = 1292.4 (4) Å^3^	0.20 × 0.15 × 0.10 mm
*Z* = 4	

### Data collection

**Table:**

Bruker APEXII CCD area-detector diffractometer	2285 independent reflections
Radiation source: fine-focus sealed tube	1977 reflections with *I* > 2σ(*I*)
graphite	*R*_int_ = 0.037
φ and ω scans	θ_max_ = 25.0°, θ_min_ = 1.7°
Absorption correction: multi-scan (*SADABS*; Sheldrick, 2003)	*h* = −5→5
*T*_min_ = 0.982, *T*_max_ = 0.991	*k* = −15→17
6296 measured reflections	*l* = −23→21

### Refinement

**Table:**

Refinement on *F*^2^	Secondary atom site location: difference Fourier map
Least-squares matrix: full	Hydrogen site location: inferred from neighbouring sites
*R*[*F*^2^ > 2σ(*F*^2^)] = 0.041	H-atom parameters constrained
*wR*(*F*^2^) = 0.118	*w* = 1/[σ^2^(*F*_o_^2^) + (0.0619*P*)^2^ + 0.1775*P*] where *P* = (*F*_o_^2^ + 2*F*_c_^2^)/3
*S* = 1.08	(Δ/σ)_max_ < 0.001
2285 reflections	Δρ_max_ = 0.15 e Å^−^^3^
155 parameters	Δρ_min_ = −0.16 e Å^−^^3^
1 restraint	Extinction correction: *SHELXL97* (Sheldrick, 2008), Fc^*^=kFc[1+0.001xFc^2^λ^3^/sin(2θ)]^-1/4^
Primary atom site location: structure-invariant direct methods	Extinction coefficient: 0.110 (8)

### Special details

**Table:**

Geometry. All e.s.d.'s (except the e.s.d. in the dihedral angle between two l.s. planes) are estimated using the full covariance matrix. The cell e.s.d.'s are taken into account individually in the estimation of e.s.d.'s in distances, angles and torsion angles; correlations between e.s.d.'s in cell parameters are only used when they are defined by crystal symmetry. An approximate (isotropic) treatment of cell e.s.d.'s is used for estimating e.s.d.'s involving l.s. planes.
Refinement. Refinement of *F*^2^ against ALL reflections. The weighted *R*-factor *wR* and goodness of fit *S* are based on *F*^2^, conventional *R*-factors *R* are based on *F*, with *F* set to zero for negative *F*^2^. The threshold expression of *F*^2^ > σ(*F*^2^) is used only for calculating *R*-factors(gt) *etc*. and is not relevant to the choice of reflections for refinement. *R*-factors based on *F*^2^ are statistically about twice as large as those based on *F*, and *R*- factors based on ALL data will be even larger.

### Fractional atomic coordinates and isotropic or equivalent isotropic displacement parameters (Å^2^)

**Table:**

	*x*	*y*	*z*	*U*_iso_*/*U*_eq_	
C1	1.0716 (4)	−0.06793 (13)	0.67868 (10)	0.0771 (5)	
H1A	1.1839	−0.0662	0.7202	0.116*	
H1B	1.2038	−0.0808	0.6419	0.116*	
H1C	0.9220	−0.1149	0.6812	0.116*	
C2	0.7645 (3)	0.02789 (10)	0.61180 (6)	0.0485 (4)	
C3	0.6201 (3)	0.11655 (9)	0.60495 (6)	0.0429 (3)	
C4	0.6275 (3)	0.19623 (9)	0.64214 (6)	0.0428 (3)	
C5	0.4354 (3)	0.25896 (9)	0.60761 (6)	0.0440 (3)	
C6	0.3161 (3)	0.21461 (9)	0.55064 (6)	0.0422 (3)	
C7	0.1103 (3)	0.24441 (10)	0.49502 (7)	0.0504 (4)	
H7A	−0.0305	0.2888	0.5126	0.060*	
H7B	−0.0030	0.1920	0.4789	0.060*	
C8	0.2772 (4)	0.28646 (14)	0.43641 (8)	0.0753 (5)	
H8A	0.1370	0.3043	0.4018	0.113*	
H8B	0.4146	0.2425	0.4184	0.113*	
H8C	0.3857	0.3393	0.4519	0.113*	
C9	0.3744 (3)	0.35270 (11)	0.62967 (7)	0.0548 (4)	
C10	0.1230 (6)	0.49089 (14)	0.60468 (13)	0.1063 (8)	
H10A	0.0056	0.5182	0.5692	0.159*	
H10B	0.2992	0.5270	0.6126	0.159*	
H10C	0.0071	0.4882	0.6453	0.159*	
C11	0.8015 (3)	0.21297 (11)	0.70625 (7)	0.0530 (4)	
H11A	0.8638	0.2765	0.7077	0.064*	
H11B	0.9803	0.1754	0.7062	0.064*	
C12	0.6215 (4)	0.19160 (16)	0.76897 (8)	0.0802 (6)	
H12A	0.7415	0.2033	0.8085	0.120*	
H12B	0.5628	0.1284	0.7683	0.120*	
H12C	0.4463	0.2296	0.7698	0.120*	
N1	0.4307 (2)	0.12986 (7)	0.55029 (5)	0.0439 (3)	
H1N	0.3918	0.0897	0.5207	0.053*	
O1	0.2088 (3)	0.39946 (8)	0.58509 (7)	0.0858 (4)	
O2	0.4557 (3)	0.38626 (9)	0.68180 (7)	0.0867 (4)	
O3	0.9285 (3)	0.01959 (7)	0.66768 (5)	0.0619 (3)	
O4	0.7364 (3)	−0.03336 (7)	0.57103 (5)	0.0725 (4)	

### Atomic displacement parameters (Å^2^)

**Table:**

	*U*^11^	*U*^22^	*U*^33^	*U*^12^	*U*^13^	*U*^23^
C1	0.0872 (12)	0.0737 (11)	0.0700 (11)	0.0164 (9)	−0.0229 (9)	0.0148 (9)
C2	0.0557 (8)	0.0537 (8)	0.0361 (7)	−0.0007 (6)	−0.0071 (6)	0.0027 (6)
C3	0.0452 (7)	0.0510 (8)	0.0324 (6)	−0.0027 (6)	−0.0039 (5)	0.0028 (5)
C4	0.0428 (7)	0.0518 (7)	0.0337 (6)	−0.0065 (5)	0.0008 (5)	−0.0004 (5)
C5	0.0448 (7)	0.0501 (7)	0.0370 (7)	−0.0040 (6)	0.0018 (5)	−0.0018 (5)
C6	0.0426 (7)	0.0485 (7)	0.0356 (7)	−0.0011 (5)	0.0022 (5)	0.0011 (5)
C7	0.0499 (7)	0.0581 (8)	0.0430 (7)	0.0065 (6)	−0.0060 (6)	−0.0012 (6)
C8	0.0784 (11)	0.0967 (13)	0.0507 (9)	0.0129 (10)	−0.0038 (8)	0.0251 (9)
C9	0.0604 (9)	0.0543 (8)	0.0497 (8)	−0.0030 (7)	0.0003 (7)	−0.0054 (7)
C10	0.144 (2)	0.0585 (11)	0.1155 (18)	0.0297 (12)	−0.0188 (16)	−0.0147 (12)
C11	0.0521 (8)	0.0635 (9)	0.0432 (8)	−0.0057 (7)	−0.0091 (6)	−0.0072 (6)
C12	0.0775 (12)	0.1278 (17)	0.0351 (8)	−0.0093 (11)	−0.0089 (7)	−0.0013 (9)
N1	0.0515 (7)	0.0469 (6)	0.0332 (6)	−0.0012 (5)	−0.0062 (5)	−0.0037 (4)
O1	0.1210 (11)	0.0572 (7)	0.0788 (8)	0.0254 (7)	−0.0276 (8)	−0.0122 (6)
O2	0.1163 (11)	0.0700 (8)	0.0733 (8)	0.0097 (7)	−0.0253 (7)	−0.0288 (6)
O3	0.0770 (7)	0.0614 (7)	0.0468 (6)	0.0075 (5)	−0.0213 (5)	0.0043 (5)
O4	0.1030 (9)	0.0579 (7)	0.0560 (7)	0.0185 (6)	−0.0278 (6)	−0.0112 (5)

### Geometric parameters (Å, °)

**Table:**

C1—O3	1.446 (2)	C7—H7B	0.9700
C1—H1A	0.9600	C8—H8A	0.9600
C1—H1B	0.9600	C8—H8B	0.9600
C1—H1C	0.9600	C8—H8C	0.9600
C2—O4	1.2109 (17)	C9—O2	1.1959 (18)
C2—O3	1.3270 (16)	C9—O1	1.335 (2)
C2—C3	1.453 (2)	C10—O1	1.444 (2)
C3—C4	1.3779 (19)	C10—H10A	0.9600
C3—N1	1.3816 (16)	C10—H10B	0.9600
C4—C5	1.4266 (19)	C10—H10C	0.9600
C4—C11	1.5024 (18)	C11—C12	1.517 (2)
C5—C6	1.4017 (17)	C11—H11A	0.9700
C5—C9	1.464 (2)	C11—H11B	0.9700
C6—N1	1.3404 (17)	C12—H12A	0.9600
C6—C7	1.4935 (18)	C12—H12B	0.9600
C7—C8	1.514 (2)	C12—H12C	0.9600
C7—H7A	0.9700	N1—H1N	0.8457
			
O3—C1—H1A	109.5	C7—C8—H8C	109.5
O3—C1—H1B	109.5	H8A—C8—H8C	109.5
H1A—C1—H1B	109.5	H8B—C8—H8C	109.5
O3—C1—H1C	109.5	O2—C9—O1	121.51 (15)
H1A—C1—H1C	109.5	O2—C9—C5	125.79 (15)
H1B—C1—H1C	109.5	O1—C9—C5	112.69 (13)
O4—C2—O3	122.75 (13)	O1—C10—H10A	109.5
O4—C2—C3	123.61 (12)	O1—C10—H10B	109.5
O3—C2—C3	113.64 (12)	H10A—C10—H10B	109.5
C4—C3—N1	108.11 (12)	O1—C10—H10C	109.5
C4—C3—C2	134.03 (12)	H10A—C10—H10C	109.5
N1—C3—C2	117.86 (11)	H10B—C10—H10C	109.5
C3—C4—C5	106.00 (11)	C4—C11—C12	112.50 (12)
C3—C4—C11	126.77 (13)	C4—C11—H11A	109.1
C5—C4—C11	127.23 (12)	C12—C11—H11A	109.1
C6—C5—C4	108.15 (12)	C4—C11—H11B	109.1
C6—C5—C9	127.00 (13)	C12—C11—H11B	109.1
C4—C5—C9	124.85 (12)	H11A—C11—H11B	107.8
N1—C6—C5	106.78 (11)	C11—C12—H12A	109.5
N1—C6—C7	119.92 (12)	C11—C12—H12B	109.5
C5—C6—C7	133.28 (13)	H12A—C12—H12B	109.5
C6—C7—C8	112.23 (12)	C11—C12—H12C	109.5
C6—C7—H7A	109.2	H12A—C12—H12C	109.5
C8—C7—H7A	109.2	H12B—C12—H12C	109.5
C6—C7—H7B	109.2	C6—N1—C3	110.97 (11)
C8—C7—H7B	109.2	C6—N1—H1N	124.6
H7A—C7—H7B	107.9	C3—N1—H1N	124.4
C7—C8—H8A	109.5	C9—O1—C10	116.33 (15)
C7—C8—H8B	109.5	C2—O3—C1	116.55 (12)
H8A—C8—H8B	109.5		

### Hydrogen-bond geometry (Å, °)

**Table:**

*D*—H···*A*	*D*—H	H···*A*	*D*···*A*	*D*—H···*A*
N1—H1N···O4^i^	0.85	2.07	2.8773 (15)	160.

Symmetry codes: (i) −*x*+1, −*y*, −*z*+1.
